# *Ligilactobacillus salivarius* PS2 Supplementation during Pregnancy and Lactation Prevents Mastitis: A Randomised Controlled Trial

**DOI:** 10.3390/microorganisms9091933

**Published:** 2021-09-11

**Authors:** Esther Jiménez, Susana Manzano, Dietmar Schlembach, Krzysztof Arciszewski, Rocio Martin, Kaouther Ben Amor, Mieke Roelofs, Jan Knol, Juan Miguel Rodríguez, Michael Abou-Dakn, PREMIUM Study Group

**Affiliations:** 1 Probisearch SLU, 28760 Tres Cantos, Spain; susana.manzano@probisearch.com; 2 Department of Nutrition and Food Science, Complutense University of Madrid, 28040 Madrid, Spain; jmrodrig@ucm.es; 3 Clinic for Obstetrics, Vivantes Hospital Group, Neukölln, 12351 Berlin, Germany; drschlembach@gmx.net; 4 Poliklinika Ginekologiczno-Położnicza Sp. z o.o. Sp. K, 15-435 Białystok, Poland; krzysztof@arciszewski.pl; 5 Danone Nutricia Research, 3584 CT Utrecht, The Netherlands; rocio.martin@danone.com (R.M.); kaouther.benamor@danone.com (K.B.A.); Mieke.Roelofs@danone.com (M.R.); jan.knol@danone.com (J.K.); 6 Laboratory of Microbiology, Wageningen University, 6708 WE Wageningen, The Netherlands; 7 Berlin Center for Diabetes and Pregnancy, Department of Obstetrics and Gynecology, St. Joseph Hospital, 12101 Berlin, Germany; Michael.Abou-Dakn@sjk.de

**Keywords:** breastfeeding support, mastitis, prevention, probiotics, *Lactobacillus salivarius*, *Ligilactobacillus salivarius*, pregnancy, lactation, randomized controlled trial, nutritional interventions

## Abstract

Mastitis is considered one of the main reasons for unwanted breastfeeding cessation. This study aimed to investigate the preventive effect of the probiotic strain *Ligilactobacillus salivarius* PS2 on the occurrence of mastitis in lactating women. In this multicountry, multicenter, randomized, double-blind, placebo-controlled trial, 328 women were assigned to the probiotic or the placebo group. The intervention started from the 35th week of pregnancy until week 12 post-partum. The primary outcome was the incidence (hazard) rate of mastitis, defined as the presence of at least two of the following symptoms: breast pain, breast erythema, breast engorgement not relieved by breastfeeding, and temperature > 38 °C. The probability of being free of mastitis during the study was higher in the probiotic than in the placebo group (*p* = 0.022, Kaplan–Meier log rank test) with 9 mastitis cases (6%) vs. 20 mastitis cases (14%), respectively. The hazard ratio of the incidence of mastitis between both study groups was 0.41 (0.190–0.915; *p* = 0.029), indicating that women in the probiotic group were 58% less likely to experience mastitis. In conclusion, supplementation of *L. salivarius* PS2 during late pregnancy and early lactation was safe and effective in preventing mastitis, which is one of the main barriers for continuing breastfeeding.

## 1. Introduction

Mastitis is defined as “an inflammatory condition of the breast, which may or may not be accompanied by infection” [1], and it is considered to be one of the reasons to stop breastfeeding [2,3,4].

The worldwide reported mastitis incidence varies, but it is estimated to generally affect between 10% and 33% of lactating women [1]. A recent review indicated that approximately one out of four breastfeeding women has at least one episode of mastitis during the first 26 weeks postpartum [5]. This wide variation among studies is most likely due to lack of a standard medical definition and well-defined diagnostic criteria. A generally accepted diagnosis is the assessment of clinical symptoms consisting of flu-like symptoms, breast pain and a clinical assessment of the breast for signs of inflammation, erythema, fever, and engorgement [6]. Other analytical approaches based on bacteriological assessment of human milk samples have been seldom used, mainly due to the absence of standardized sampling, analysis procedures and lack of consensus on the etiology of mastitis [7,8].

To support breastfeeding duration, different strategies to prevent mastitis have been reported. These include education and advice on best practices of breastfeeding to lactating women by their health care professionals, antibiotic use, topical ointment and alternative therapies [9]. Yet, a Cochrane review did not find enough proof to support such practices [10]. Therefore, with a lack of substantiation for effective preventive strategies, lactational mastitis is often treated with antibiotics [11]. Although narrow-spectrum antibiotics (ie., Flucoxacillin) are recommended to treat the major etiological agents of infectious mastitis (*Staphylococcus aureus*), broad spectrum antibiotics are also frequently used [12]. However, it is well acknowledged that antibiotics use for lactational mastitis may have numerous health consequences, such as the burden of antibiotic resistance and disturbance of both the gastrointestinal and human milk microbiota, which can result in undesired biofilm formation, and gastrointestinal, urogenital or oral infections [13,14,15]. Furthermore, medical doctors and families are skeptical about using antibiotics during lactation due to the possible exposure of the infant to these medication during breastfeeding. With the emerging scientific knowledge and interest on the human milk microbiome, probiotics have been proposed as a potential alternative for the management of mastitis [16] and several studies have indeed provided evidence for the potential use of probiotics for mastitis treatment [17,18,19], and prevention [20,21].

*Ligilactobacillus salivarius* PS2, formerly classified as *Lactobacillus salivarius* PS2 [22], a probiotic strain isolated from the milk of a healthy woman, was tested in clinical trials for its potential in mastitis treatment. In a pilot study, Espinosa-Martos et al. [23] showed that oral administration of *L. salivarius* PS2 resulted in changes in microbiological and immunological biomarkers related to mastitis [23]. In another clinical trial, supplementation with *L. salivarius* PS2 during late pregnancy of women with mastitis during a previous lactation period was efficient to prevent infectious mastitis [20]. In this context, the objective of this study was to evaluate, for the first time, if oral administration of *L. salivarius* PS2 during both late pregnancy and lactation was able to reduce the risk of developing mastitis in lactating women.

## 2. Materials and Methods

### 2.1. Study Design and Ethical Considerations

This multicountry, multicenter, randomized, double-blinded, placebo-controlled trial was conducted between October 2014 and October 2017 in hospitals and primary health centers in Spain, Poland, Germany, and Austria. Participating centers obtained approval from their independent local Ethical Review Boards. Written informed consent was obtained from each participant before the start of the study. The study was conducted according to Good Clinical Practice (GCP) defined by the International Council for Harmonization of Technical Requirements for Pharmaceuticals for Human Use (ICH), in compliance with the Declaration of Helsinki developed by the ‘World Medical Association Declaration of Helsinki’ (64th WMA General Assembly, Fortaleza, Brazil, October 2013). The study was registered in the Dutch Trial Register with identifier: NTR 4388 on 7 January 2014 (new ID in Dutch Trial Register: NL4243).

### 2.2. Study Participants and Study Procedure

Participants eligible for this study were required to meet the inclusion criteria of healthy pregnant women ≥ 18 years of age between the 33rd and 35th week of pregnancy, and with the intention to breastfeed. Recruitment was performed by the midwife/doctor responsible for the pregnancy care. Exclusion criteria were a pre-gravid body mass index (BMI) < 18 (underweight) or >30 (overweight), the use of probiotic supplements during the third trimester of pregnancy, an enhanced chance of premature delivery (before 37 weeks + 0 days of gestation), and current or previous severe illnesses (full list of exclusion criteria in Supplementary Table S1).

The participants had to visit the study site three times and were interviewed by phone twice according to the following schedule: at the beginning of the study (visit 1; between the 33rd and 35th week of pregnancy); within seven days after delivery (visit 2); six weeks after delivery (phone call 1), 12 weeks after delivery (visit 3), and two weeks after intake of the last supplement (phone call 2). When a woman had symptoms that could be due to mastitis, an additional visit to the study site was scheduled to confirm the diagnosis and to start medical treatment, followed up by a phone interview one week later (phone call A1), and a medical examination two weeks after diagnosis (visit A2). Visits continued weekly until remission of symptoms. Diagnosis, treatment, and evaluation of mastitis symptoms were performed at the study sites according to routine clinical procedures.

### 2.3. Intervention

The test product was a probiotic supplement packed in sealed 1 g-sachets as a freeze-dried powder. The product contained approximately 1 × 10^9^ CFU *L. salivarius* PS2 (a strain isolated from milk of a healthy woman) [20] and carrier materials (83% maltodextrin, 16% starch, and 1% calcium silicate) to sustain a good viability of the strain. Both study products were produced by DuPont (Madison, WI, USA) and provided to the sites by Danone Nutricia Research.

At visit 1, eligible women were randomly allocated to either the probiotic or the placebo group. Women in the probiotic group received a daily oral dose of ~10^9^ CFU of *L. salivarius* PS2 from the 35th week of pregnancy until week 12 after delivery. The placebo group received a placebo containing only the carrier materials and did not differ in appearance and/or taste with the probiotic supplement. 

### 2.4. Randomization

Randomization was stratified per site with block size equal to 8. Based on the order in which they entered the study, subjects were assigned a randomization number. These randomization numbers were linked to either the test product or the placebo (50% chance to be linked to either one of the products), using a computer random number generator. The details of the randomization were unknown to the investigator, the participants, the site staff, or to the study staff from Danone Nutricia Research (except from the statistician who was responsible for generating the randomization sequence, and the supply manager who needed to be unblinded in order to label the study products). The randomization sequence was generated using SAS, version 9.2.

### 2.5. Data Collection

At visit 1, demographic characteristics and relevant medical history were recorded by the doctor/midwife. The investigators were asked to record at least the following data: history of lactation and mastitis after previous pregnancies, and any previous caesarean section (C-section) or planned C-section for the current pregnancy. The relevance of the other medical events was based on the investigators’ clinical judgment.

Participants recorded daily the intake of the study product for the evaluation of compliance. Data on breastfeeding, such as number of feedings and type of feeding (exclusive and/or mixed feeding) was also reported. For mastitis diagnosis, the presence of local breast symptoms (pain, redness, and breast engorgement) and fever (defined as body temperature > 38 °C) was recorded. In case of mastitis symptoms, diagnosis was confirmed by the doctor or midwife. The severity of mastitis was evaluated using the mastitis severity index described earlier by Kvist et al., [24]. This index has a range 0–19 and includes scales for erythema (0–4), breast tension (0–5) and pain (0–10). The scales are defined as erythema (0 = no redness, 1 = slight redness in a limited area, 2 = redness in a limited area, 3 = bright red in a limited area, 4 = bright red over most of the breast), breast tension (0 = soft, no tension, 1 = firm, no tenderness, 2 = tense, but not uncomfortable, 3 = tense and uncomfortable, 4 = tense and painful, 5 = very tense and very painful) and pain (0 = no pain to 10 = extreme pain). Data about the use of medication related to mastitis such as antibiotics and anti-inflammatory drugs were also collected.

Adverse events (AEs) were coded according to the medical dictionary for regulatory activities (MedDRA version 17.1), including five different levels of hierarchy, from very specific to very general. System organ classes (SOC, the most general level), and the preferred term (a distinct descriptor for a symptom) were used to code the AE data into an internationally standardized medical terminology. In addition, a questionnaire was used to evaluate gastrointestinal symptoms (abdominal distension, flatulence, diarrhea, constipation) with a four-point scale (absent, mild, moderate, severe). Frequency of bowel movements and consistency of stools were recorded with a 5-point scale (1: watery, 2: soft, pudding like, 3: soft, formed, 4: dry, formed, 5: dry, hard pellets).

### 2.6. Study Outcomes

The primary outcome was the incidence (hazard) rate of mastitis. In this study, mastitis was defined as having at least two of the following symptoms: breast pain, breast erythema, breast engorgement that was not relieved by breastfeeding, and maternal temperature above 38 °C [6] (Cusack & Brennan, 2011). Secondary and exploratory parameters included: (i) complete or partial discontinuation of breastfeeding, (ii) recurrent episodes of mastitis, (iii) duration of mastitis episodes, (iv) fever due to mastitis, (v) mastitis severity index, (vi) mastitis pain score, and (vii) medication to treat mastitis. Safety and tolerance parameters included AEs and SAEs, concomitant medication and gastrointestinal symptoms.

### 2.7. Determination of Sample Size

Existing data from a prospective cohort study [25] on the occurrence of mastitis was used in combination with an assumed total incidence in the control group of 30% to estimate the percentage of mastitis-free women in the control group over time (84.1% at 4 weeks, 77.8% at 8 weeks, and 70% at 12 weeks). For the probiotic group, a 50% decrease in mastitis and linear occurrence of mastitis over time was used to estimate the percentage of mastitis-free women (95% at 4 weeks, 90% at 8 weeks, 85% at 12 weeks). Using such assumptions, a significance level (α) of 0.05, a power of 80%, and a sample size of 232 (116 for each of the two groups) were assumed to be enough to detect a statistically significant difference in the mastitis-free survival curves between the probiotic group and the control group using a Log-rank test (SAS version 9.2). Assuming a dropout rate of approximately 20%, the total number of subjects to be included in the study was calculated to be 300.

### 2.8. Statistical Analysis

Survival analysis was carried out to evaluate the primary outcome parameter i.e., incidence (hazard) rate of mastitis. As a first step, Kaplan–Meier estimators were used for assessing the probability of being free of mastitis at a specified time. The Log-rank test was used for comparing the survival curves of both groups. This approach tests whether the probability of having a mastitis event in any group is equal (at any time-point during the study). A *p*-value ≤ 0.05 indicated that the mastitis rates differed between groups at one or more time-points during the study (i.e., the probability of being free of mastitis is different). As a second step, Cox proportional hazards models were used to compare the hazard functions, adjusting for age at delivery and site.

Outcome parameters with a continuous distribution were analyzed using linear models and those with discrete distribution (dichotomous variables, ordered categorical variables, and counts) with generalized linear models or non-parametric statistics. Transformations were performed to improve distribution.

The mITT (modified Intention-To-Treat) included randomized women who were deemed eligible after randomization and who participated in the study at least until giving birth. As women were only at risk of developing mastitis after delivery, women for whom no post-delivery data were available were excluded. 

The mITT data set was used for the analysis of the primary outcome, the secondary parameters, and the exploratory parameters. The AST (All-Subjects-Treated) dataset was used for the safety analysis.

## 3. Results

### 3.1. Study Population/Participants

**Figure 1:**
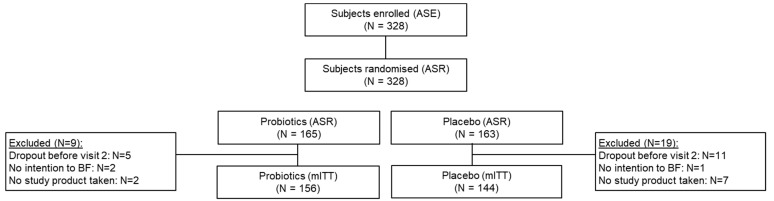
Flow chart of women participating in the study. ASR, All Subjects Randomized; mITT (modified Intention-To-Treat), AST, All Subjects Treated.

From October 2014 to October 2017, a total of 328 women were enrolled and randomized, of which 142 were included from Spain (43.3%), 115 in Poland (35.1%), 40 in Germany (12.2%) and 31 in Austria (9.4%). In total, 28 randomized subjects were excluded from the mITT population due to reasons specified in Figure 1.

Women were well balanced over the two study groups in the mITT population with respect to the baseline characteristics, including maternal age at inclusion, pre-gravid BMI, number of previous children, previous mastitis, type of delivery. Regarding the infants, gestational age and birth weight were not different between the study groups (Table 1). Participants adhered to product intake very well throughout the study and only 30 women had product compliance less than 85% in the mITT population (15 in probiotic group and 15 in placebo group). 

### 3.2. Occurrence of Mastitis 

**Figure 2:**
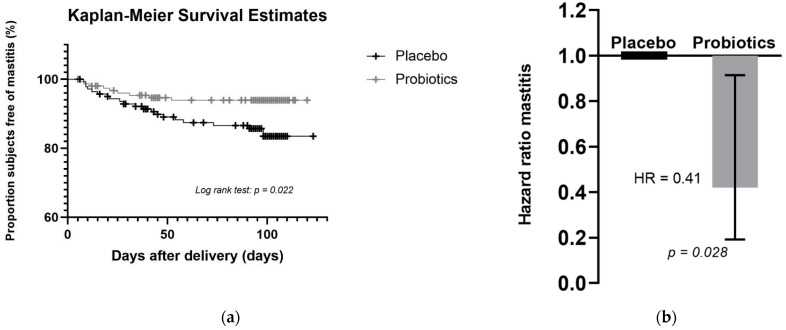
Effect of *L. salivarius* PS2 on the risk of developing mastitis: (**a**) Kaplan–Meier plot showing the percentage of subjects free of mastitis in the probiotics and placebo groups; (**b**) Hazard ratio (with 95% CI) for treatment determined by Cox’s PH regression model showing that subjects in the probiotic group were 59% less likely to develop mastitis.

In total, 29 (9.7%) women of the mITT population reported mastitis, of which 9 (6%) were in the probiotic group and 20 (14%) were in the placebo group. Figure 2a shows the Kaplan–Meier curves illustrating a higher probability of being free of mastitis for the probiotic group compared to the placebo group. The comparison between the two survival curves was performed using the log-rank test, and indeed the difference between mastitis-free survival curves in probiotic group and placebo group was statistically significant (*p* = 0.022).

When using the Cox’s PH regression model stratified by site and including treatment group and maternal age at delivery as explanatory variables, there was a decreased risk of mastitis in the probiotics treatment group as compared to the placebo group (hazard ratio = 0.41; 95% CI: 0.19–0.91, *p* = 0.028) (Figure 2b). The exponential of the coefficient for treatment, 0.41, indicates that a subject who received the probiotic supplement was 59% less likely to experience mastitis than one who received the placebo supplement (95% CI: 9–81%). A fully adjusted Cox PH regression model was applied as sensitivity analysis by adding the following potential covariates to the model and using backward selection to assess robustness of the results: use of antibiotics, type of delivery, number of previous breastfed infants, duration of breastfeeding, breastfeeding cessation, previous mastitis, and duration of study product intake before delivery. The final model after backward elimination retained only the treatment effect and confirmed a decreased risk of mastitis for the subjects in the probiotic group as compared to subjects in the placebo group (hazard ratio = 0.42; 95% CI: 0.19–0.91; *p* = 0.029) (Supplementary Table S2).

### 3.3. Secondary and Exploratory Outcome 

Regarding breastfeeding cessation, 22 women in the mITT population (16 in the probiotic group and 6 in the placebo group) stopped breastfeeding during the study, among them 16 who prematurely terminated the study (11 in the probiotic group and 5 in the placebo group). Mastitis was reported for only one woman (probiotic group) who stopped breastfeeding. During the 12 weeks study period, the median (Q1, Q3) duration of exclusive breastfeeding was 77 (41, 83) days and 76 (30, 83) days in the placebo and probiotic group, respectively, (Supplementary Table S3).

In total, 31 episodes of mastitis (22 episodes in the placebo group and 9 in the probiotic group) were reported among the 29 women in the mITT population who developed mastitis during the study. Since the number of recurrent episodes of mastitis was extremely low (2) and only happened in the placebo group, none of the previously planned analyses were performed on this parameter. Among the 22 episodes of mastitis reported in the placebo group, 6 (27.3%) included fever as a symptom, compared to 5 out of 9 episodes (55.6%) in the probiotic group (*p* = 0.369). The median (Q1, Q3) days duration of mastitis episodes were 15 (11.0, 22.0) and 16 (10.0, 18.0) in the placebo and probiotic group, respectively, (*p* = 0.928) (Table 2). 

Women who suffered from mastitis scored their breast pain in a scale ranging from 0 (no pain) to 10 (extreme pain). At the first diagnose (visit A1), the median (Q1, Q3) breast pain score was 7.0 (3.0, 8.0) and 6.0 (6.0, 7.0) in the placebo and probiotic group, respectively (*p* = 0.564). A decrease in the median intensity of breast pain was observed during the study period in both the placebo and probiotic group (Table 3). Although the difference between both groups was not statistically significant, notably a decrease in the median (Q1, Q3) pain score was observed in the probiotics compared to the placebo group −3.0 (−6.0, −2.0), −6.0 (−7.0, −5.0), respectively, (*p* = 0.098) (Table 3). The mastitis severity index, resulting from the sum of scores for breast pain, erythema, and breast tension, decreased during the intervention in both groups, as illustrated in Table 3. The median (Q1, Q3) change from visit A1 to visit A2 was −8.0 (−11.0, −5.0) in the probiotic compared to −11.5 (−12.0, −9.5) in the placebo group (*p* = 0.163). 

In the probiotic group, 4 women (44.4%) received antibiotics compared to 16 women (72.7%) in the placebo group (*p* = 0.217). The percentage of women who used anti-inflammatory drugs was 77.8% in the probiotic group compared to 68.2% in the placebo group (*p* = 0.689).

### 3.4. Safety and Tolerance Evaluation 

The probiotic product was well tolerated, and both groups showed a similar safety profile, based on the number and severity of AEs and gastrointestinal (GI) symptoms. In total, 63.7% of subjects in the placebo group as compared to 59.5% in the probiotic group reported at least one AE. The AEs related to pregnancy, puerperium and perinatal conditions were higher in the placebo group (25.5%) than in the probiotic group (13.5%) (*p* = 0.007). All the reported AEs in the probiotic group were not related to the study product. SAE was reported in 5.1% of subjects in the placebo group and 1.2% subjects in the probiotic group (*p* = 0.057). None of the SAE was related to the study product. The type of SAEs was generally associated with pregnancy, delivery, and lactation and, therefore, expected in these subjects’ population. The type of reported medication was in line with the type of medical histories and AEs reported during the study. There were no differences in reported GI symptoms and stool characteristics (frequency and consistency) between the probiotic and placebo group.

## 4. Discussion

This present study investigated the potential effect of oral administration of the probiotic *L. salivarius* PS2 during late pregnancy and early lactation on the prevention of mastitis incidence in lactating women. A total of 29 women (9.7%) developed mastitis, this incidence is in line with data published by the WHO, which reported incidences varying from a 10% to 33% [1]. For the sample size calculation of this study, a higher incidence was considered, as most women were expected to be enrolled in Spain, where the incidence of mastitis is reported to be relatively high (24–41%) [20,21]. Following the 12 week-intervention, women in the probiotic group were 59% less likely to experience mastitis compared to those in the placebo group. To our knowledge, this is the first study where a specific probiotic strain has been shown to reduce the risk of mastitis in lactating women, during late pregnancy and the first 12 weeks of lactation. More recently, Fernández et al. [20] investigated the efficacy of the same probiotic strain *L. salivarius* PS2 during the last 10 weeks of pregnancy in women with previous history of lactational mastitis. They reported a statistically significant decrease in lactational mastitis rate between both groups as 57% of the women in the placebo group experienced mastitis compared to 25% in the probiotic group [20]. In another preventive study, Hurtado et al., showed that supplementation of *L. fermentum* CECT5716 to healthy lactating women resulted in a significant decrease in the incidence of mastitis given that 19.7% of women in the control group while 11.5% in the probiotic group experienced mastitis [21]. However, in the study from Hurtado et al., the dropout rate was rather high (53.4%) and mastitis incidence was reported only in the completers without providing baseline information on subjects who dropped out. This would have impacted the randomization and introduced selection bias into the analysis. In our study, the dropout rate in the probiotics and the placebo group for the total study period was comparable (22.1% and 22.9%, respectively). Before delivery (Visit 2), more subjects dropped out the study in the placebo group. None of the early terminations were reported as being due to an adverse event. Other clinical studies demonstrated the potential treatment effect of different Lactobacillus strains in women who already had developed mastitis [17,18,23]. 

An important clinical symptom in lactational mastitis is breast pain [26]. Our data revealed that the pain score and the mastitis severity index were low in the probiotic group, although they did not reach statistical significance between both intervention groups. Notably, another study using the same probiotic strain reported less severe breast pain in the *L. salivarius* PS2 group than in the control group; additionally, all women with more intense breast pain were in the control group [20]. The beneficial effect of *L. salivarius* PS2 on breast pain has been supported by the findings of Vazquez-Fresno et al. [27], showing that women suffering mastitis treated with *L. salivarius* PS2 stopped the intake of ibuprofen and acetaminophen, two drugs used to control mastitis associated pain [27]. Considered together, these results suggest that *L. salivarius* PS2 may help alleviate mastitis-related breast pain, but our study did not have enough power due to the low incidence of mastitis reported in our cohort (9.7% vs. 41% found by [20]) to be statistically significant. 

Mastitis has been reported as a common condition affecting women and leading to early cessation of breastfeeding and shorter breastfeeding duration [28]. 

This could not be confirmed in our study most probably due to the low incidence of mastitis, high breastfeeding rates during the study period, and insufficient long-term follow-up to assess the impact on the duration of full breastfeeding. One limitation of this study is, indeed, the small sample size of mastitis cases (29 subjects), which is probably related to the well-defined, strict criteria that were used. 

Mastitis can jeopardize breastfeeding, but also the consequent use of antibiotic during the perinatal period is of great concern. Broad spectrum antibiotics are used to treat mastitis because of the increase of MRSA isolates and the increasing involvement of other bacterial species [29,30], a fact that might contribute to the worldwide emergence of antibiotic resistances among clinically relevant bacteria [31,32]. In our study, the use of antibiotics by women who suffered mastitis was not statistically different in the probiotic group (44% of women suffering mastitis) compared to the 72.7% in the placebo group (*p* = 0.217). Even though the data on antibiotic use did not show a statistically significant difference between both groups, this finding is of clinical relevance and should be confirmed in a future well-powered study. 

Mastitis is an inflammation of the mammary gland and its diagnosis has been commonly based on clinical symptoms, including flu-like symptoms, fever, breast pain and any signs of inflammation. However, it has been recently proposed that analysis of the bacterial load and, more specifically, the staphylococcal bacterial load are key criteria in the determination and characterization of mastitis [33]. It is well accepted that mastitis is associated with an unbalanced microbiota composition of breast milk known as dysbiosis. Recent studies using next-generation sequencing have revealed a reduced microbial diversity of the human milk microbiota of women with mastitis, with outgrowth of opportunistic pathogenic with *Staphylococcus aureus* being identified as the main etiological agents and decrease of commensal bacteria [33,34,35,36,37]. In line with these results, multi-omics-based analysis of human milk may offer a rapid and accurate tool for diagnostic of mastitis, determination of its etiology and endorsement of an efficient management strategy for this condition. Several probiotic strains have proven to successfully restore the microbial balance in human milk by reducing staphylococcal load, which is also correlated with improved symptoms such as breast pain score [19,20]. The mechanisms underlying these effects are still to be elucidated. It is suggested to be linked to the immune-modulatory and anti-inflammatory properties of probiotic strains and potential transfer of the probiotic strain from the maternal gut to the mammary glands via the entero-mammary pathway [38,39,40]. *L. salivarius* PS2 was successfully used for the treatment of mastitis in a previous pilot trial [23]. In that study, the administration of *L. salivarius* PS2 led to significant changes in several milk microbiological, bio-chemical, and immunological parameters. They included a reduction in bacterial and leukocyte counts and in the concentration of interleukin (IL)-8; an increase in the concentrations of immunoglobulin (Ig)E, IgG3, epidermal growth factor (EGF) and IL-7, and a modification of the milk electrolyte profile. In addition, it resulted in a decrease in the leukocyte counts and concentration of some oxidative stress parameters in blood samples. The metabolomics analysis of urine samples from participants also revealed a reinforcement of the mammary epithelial barrier leading to a decrease in the presence of lactose after the treatment [27]. Additionally, a transcriptomic analysis of milk samples from the same cohort revealed that the intake of *L. salivarius* PS2 positively modified the expression of some inflammatory and cell-growth related genes in the milk somatic cells [41]. Globally, such studies provided some clues on the mechanisms explaining the efficacy of *L. salivarius* PS2 in treating or preventing mastitis. 

Our study further demonstrated that the probiotic strain *L. salivarius* PS2 was safe and well tolerated. In fact, women from the probiotic group suffered significantly less AEs associated to pregnancy, puerperium and perinatal conditions than women from the placebo group. All the reported SAEs were found not to be related to the study products. Furthermore, the probiotic strain used in this study, *L. salivarius* PS2, was isolated from the breast milk of healthy women and, thus, fulfils the criteria recommended for human probiotics: human origin, a history of safe use and adaptation to the host. 

Lactational mastitis is a common, painful condition affecting women, which significantly impacts breastfeeding duration and frequency [28]. Current treatment largely involves the use of antibiotic; a safe alternative to antibiotic therapy to prevent lactational mastitis occurrence would be preferred. To our knowledge, this is the first randomized, double-blinded, placebo-controlled trial showing the efficacy of a probiotic strain isolated from human milk, *L. salivarius* PS2, in reducing the risk of mastitis in healthy pregnant and lactating women. It is also the first time that the beneficial effect of probiotics to reduce the risk of mastitis has been demonstrated not only in subjects from Spain, but also in subjects from Poland, Germany and Austria. This study contributes to the growing evidence that probiotics offer an effective nutritional strategy, both as prophylactics and therapeutics for lactational mastitis. More research is needed to reveal how probiotics work to treat and prevent mastitis, and to confirm the impact on breastfeeding outcome.

## 5. Conclusions

This study succeeded in meeting the primary objective by demonstrating a preventive effect of supplementation with *L. salivarius* PS2 during late pregnancy and early lactation on the occurrence of mastitis in healthy breastfeeding women. As a result, the use of this strain has the potential for supporting or extending breastfeeding. Moreover, the use of this product showed no safety concerns regarding the occurrence of AEs and SAEs events.

## Figures and Tables

**Table 1:** Baseline demographic and clinical characteristics of the mITT population (mothers and infants).

	Placebo (N = 144)	Probiotics (N = 156)
Age at inclusion (years) ^†^	33.0 (30.0–36.0)	33.0 (30.0–36.0)
Pre-gravid BMI (kg/m^2^) ^†^	21.8 (20.4–24.0)	22.3 (20.3–24.4)
Number of previous children, *n* (%)		
0	67 (46.5%)	64 (41.0%)
1	57 (39.6%)	66 (42.3%)
2	15 (10.4%)	24 (15.4%)
≥3	5 (3.5%)	2 (1.3%)
Previous Mastitis, *n* (%)		
No	124 (86.1%)	140 (89.7%)
Yes	20 (13.9%)	16 (10.3%)
Infant birth weight (g) ^†^	3385.0 (3095.0–3652.5)	3367.5 (3144.5–3617.5)
Gestational age, weeks ^†^	40.4 (39.4–41.1)	40.5 (39.9–41.1)
Type of birth		
C-section, *n* (%)	59 (41.0%)	58 (37.2%)
Vaginal, *n* (%)	85 (59.0%)	98 (62.8%)
Sex of the infant		
Female, *n* (%)	70 (48.6%)	73 (46.8%)
Male, *n* (%)	74 (51.4%)	83 (53.2%)

^†^ data are reported in Median (Q1–Q3).

**Table 2:** Mastitis characteristics and medication use (mITT).

	Placebo (N = 22)	Probiotic (N = 9)
Fever due to mastitis		
Absent *n* (%)	13 (59.1%)	4 (44.4%)
Present *n* (%)	6 (27.3%)	5 (55.6%)
Unspecified *n* (%)	3 (13.6%)	0 (0.0%)
Duration of mastitis episodes, days ^†^	15.0 (11.0, 22.0)	16.0 (10.0–18.0)
Antibiotic use		
Yes *n* (%)	16 (72.7%)	4 (44.4%)
No *n* (%)	6 (27.3%)	5 (55.6%)
Anti-inflammatory drugs		
Yes, *n* (%)	15 (68.2%)	7 (77.8%)
No *n* (%)	7 (31.8%)	2 (22.2%)
Other		
Yes *n* (%)	2 (9.1%)	2 (22.2%)
No *n* (%)	20 (90.9%)	7 (77.8%)

N = number of mastitis episodes; *n* = number of subjects per category. ^†^: data reported in median (Q1, Q3).

**Table 3:** Mastitis severity index and mastitis pain score for women diagnostic as suffering from mastitis during the study (mITT).

		Placebo (N = 22)	Probiotic (N = 9)
**Mastitis pain score**			
Visit A1	*n* (*n* miss)	19 (3)	9 (0)
	Mean (SD)	5.8 (2.3)	6.9 (1.4)
	Median (Q1, Q3)	7.0 (3.0, 8.0)	6.0 (6.0, 7.0)
Visit A2	*n* (*n* miss)	18 (4)	8 (1)
	Mean (SD)	2.2 (2.7)	1.1 (2.4)
	Median (Q1, Q3)	1.0 (0.0, 3.0)	0.0 (0.0, 1.0)
Change in mastitis pain score ^†^			
	*n* (*n* miss)	18 (4)	8 (1)
	Mean (SD)	−3.7 (2.6)	−5.9 (2.5)
	Median (Q1, Q3)	−3.0 (−6.0, −2.0)	−6.0 (−7.0, −5.0)
**Mastitis severity index**			
Visit A1	*n* (*n* miss)	19 (3)	9 (0)
	Mean (SD)	11.4 (3.3)	12.1 (1.1)
	Median (Q1, Q3)	12.0 (8.0, 15.0)	12.0 (11.0, 13.0)
Visit A2	*n* (*n* miss)	18 (4)	8 (1)
	Mean (SD)	3.9 (5.2)	2.3 (4.5)
	Median (Q1, Q3)	1.0 (0.0, 6.0)	0.5 (0.0, 2.0)
Change in mastitis severity index ^†^			
	*n* (*n* miss)	18 (4)	8 (1)
	Mean (SD)	−7.7 (4.8)	−10.0 (3.9)
	Median (Q1, Q3)	−8.0 (−11.0, −5.0)	−11.5 (−12.0, −9.5)

N = number of mastitis events; *n* = number of subjects per category, *n* miss = number of missing values per category; SD = Standard Deviation, Q1, Q3 = Quartile1, Quartile 3. Visit A1: visit at first mastitis diagnostic. Visit A2: visit 2 weeks following visit A1. ^†^ Change between visit A1 and visit A2.

## Data Availability

The data presented in this study are available on request from the corresponding author. The data are not publicly available due to privacy restrictions.

## Supplementary Materials

The following are available online at https://www.mdpi.com/article/10.3390/microorganisms9091933/s1, Supplementary Table S1. Exclusion criteria for women participating in the study; Supplementary Table S2. Hazard ratio of incidence of mastitis between study groups (mITT); Supplementary Table S3. Duration of partial (mixed with formula feeding) and exclusive breastfeeding (mITT).
