# Supplementary material for: Ligilactobacillus salivarius PS2 Supplementation during Pregnancy and Lactation Prevents Mastitis: A Randomised Controlled Trial
Source: Microorganisms. 2021 Sep 11;9(9):1933. doi: 10.3390/microorganisms9091933 (PMC8469334; doi:10.3390/microorganisms9091933)
Supplement: Supplementary file 1 [file microorganisms-09-01933-s001.zip › microorganisms-1326290-supplementary.pdf]

Supporting Information

Supporting Information Table S1. Exclusion criteria for women participating in the study.

**Exclusion criteria for women participating in the study**
1) Pre-gravid body mass index (BMI)<18 or >30;
2) Use of probiotic supplements during the third trimester of current pregnancy[1];
3) Enhanced chance of premature delivery (before 37 weeks + 0 days of gestation);
4) Current or previous illnesses which could interfere with the study, like other mammary pathologies (e.g. abscesses, Raynaud’s syndrome, breast cancer);
5) Short bowel syndrome;
6) Impaired intestinal epithelial barrier (e.g. diarrheal illness, intestinal inflammation);
7) Serious underlying disease predisposing to infection (e.g. HIV, auto-immune diabetes, multiple organ failure, malignancy, severe burns, severe acute pancreatitis);
8) Heart failure and cardiac medical history (e.g. artificial heart valve, medical history of infectious endocarditis, rheumatic fever and cardiac malformation);
9) History of aggressive immunosuppressive therapy (e.g. radiotherapy, cancer chemotherapy);
10) Traumatic injury of the gastrointestinal tract;
11) Surgery, including dental surgery, within one month prior to inclusion (Visit 1);
12) Investigator's uncertainty about the willingness/ability of the subject to comply with protocol requirements;
13) Participation in any other clinical trial within two weeks prior to entry into the study.

[1]Criterion referred to products (supplements) that are bought in the pharmacy and/or health drug store. It did not involve food product supplemented with ‘live bacteria’.

Supporting Information Table S2. Hazard ratio of incidence of mastitis between study groups (mITT)

	Placebo (N=144)	Probiotic (N=156)	P-value
Mastitis incidence			
No, n (%)	124 (86.1%)	147 (94.2%)	
Yes, n (%)	20 (13.9%)	9 (5.8%)	
Age adjusted HR (CI) †	1.000	0.415 (0.189-0.912)	0.0285
Fully adjusted HR (CI) ‡	1.000	0.352 (0.156-0.793)	0.0117
Fully adjusted HR (CI)§	1.000	0.417 (0.190-0.915)	0.0293

N=Number of subjects of the analysis population, [n]=number of subjects per category. HR=Hazard Ratio, CI=Confidence Interval

†Cox PH regression model stratified by sub-site and includes study group and age at delivery as covariates.

‡Fully adjusted Cox PH regression model, adding the following potential covariates to the model: use of antibiotics, type of delivery, number of previous breastfed infants, duration of breastfeeding, breastfeeding stop, previous mastitis, and duration of study product intake before delivery.

§Fully adjusted Cox PH regression model, adding the potential covariates and applying backward selection.

Supporting Information Table S3. Duration of partial (mixed with formula feeding) and exclusive breastfeeding (mITT)†

		**Placebo (N=144)**	**Probiotics (N=156)**	**P-value***
Duration of any breastfeeding (including mixed), days‡	n (n miss)	137 (7)	151 (5)	
	Mean (SD)	77.2 (19.6)	77.3 (19.2)	
	Median	84.0	83.0	0.765
	(Q1, Q3)	(79.0, 86.0)	(80.0, 85.0)	
	Min - Max	5.0 - 104.0	5.0 - 105.0	
Duration of exclusive breastfeeding, days§	n (n miss)	137 (7)	151 (5)	
	Mean (SD)	61.6 (29.8)	58.1 (32.6)	
	Median	77.0	76.0	0.559
	(Q1, Q3)	(41.0, 83.0)	(30.0, 83.0)	
	Min - Max	0.0 - 103.0	0.0 - 99.0	

* P-value of Mann-Whitney U-test

N=number of subjects of the analysis population, n=number of subjects per category, n miss=number of missing values per category, SD=Standard Deviation, Q1, Q3=Quartile1, Quartile 3

†) Data collection was limited to the 12-week follow-up of subjects. If subjects were still breastfeeding at the last visit this was recorded as the end of the breastfeeding period

‡ Duration of any breastfeeding (including mixed) was calculated as: date of last breastfeeding - date of delivery.

§ Duration of exclusive breastfeeding was calculated as: date of last breastfeeding – date of delivery - number of days with formula feeding.
